# Molecular Epidemiological and Serological Studies of Bovine Leukemia Virus in Taiwan Dairy Cattle

**DOI:** 10.3389/fvets.2019.00427

**Published:** 2019-12-06

**Authors:** Jui-Chun Hsieh, Chang-Yan Li, Wei-Li Hsu, Shih-Te Chuang

**Affiliations:** ^1^Department of Veterinary Medicine, College of Veterinary Medicine, National Chung Hsing University, Taichung, Taiwan; ^2^Graduate Institute of Microbiology and Public Health, College of Veterinary Medicine, National Chung Hsing University, Taichung, Taiwan

**Keywords:** bovine leukemia virus, dairy cattle, seroprevalence, proviral DNA, genotype

## Abstract

Bovine leukemia virus (BLV) infection results in a decrease in milk yield and quality, a compromise in immunity, and shortening in the longevity of cows. The current status of BLV infection of dairy cattle in Taiwan remains unclear. To evaluate BLV infection, anti-BLV gp51 antibody and proviral DNA were detected. Surprisingly, the seroprevalence of BLV at the animal and herd level was as high as 81.8% (540/660 cattle) and 99.1% (109/110 herds), respectively. Among 152 blood samples analyzed, 132 (86.8%) were detected as positive for BLV-proviral DNA. When the complete blood count (CBC) was taken into account, the white blood cell (WBC) number appears to be the factor with the highest predicted potential for BLV infection. Moreover, based on receiver operating characteristic (ROC) curve analysis, the sensitivity and specificity are 72.0 and 75.0%, respectively, when the cut-off value of the WBC was set at 10.215 K/μL. Despite the co-circulation of genotype 1 and 3 in Taiwan, genotype 1 was much more prevalent (29/30). Taken together, due to the high prevalence of BLV, the identification of risk factors for interrupting the routes of transmission of BLV are critical for the control and prevention of further BLV infection.

## Introduction

Bovine leukemia virus (BLV), classified into genus *Deltaretrovirus* of the family *Retroviridae* (1), is one of the most widespread pathogens in the dairy sector worldwide (2–5). BLV is the etiologic agent of enzootic bovine leukosis (6). Most affected animals (60–70%) remain subclinical or without hematologic signs (7). Nevertheless, accumulated evidence supports the notion that BLV infection likely leads to a shorter life-span and decreased milk yields and quality (5, 8), while also affecting immune function (9). Some infected cattle might develop persistent lymphocytosis (PL) or even malignant lymphoma (10), as indicated by an increase in circulating lymphocytes (9) or neutrophils or in total WBC counts (11), which could serve as surrogate markers for evaluations of BLV infection.

Considering that not all infected animals have developed persistent lymphocytosis, the diagnosis of BLV infection has been primarily based on the detection of circulating anti-viral antibodies (e.g., the envelope proteins gp51 and gp24) elicited by infection (12). As with all retroviruses, the integration of proviral DNA to the host genome is one of the essential steps in the BLV replication cycle. Therefore, numerous PCR-based methods were developed as highly sensitive molecular diagnosis platforms for BLV infection (13–15).

BLV surveillance results in Taiwan were documented in 1991, showing BLV seroprevalence of 8.4%, and 5.8% for samples collected in 1985 and 1986, respectively (16); however, surveillance results have not been updated since that time. Therefore, the objective of this study was to estimate the recent prevalence of BLV infection in Taiwan and molecularly characterize the BLV sequences identified in the investigation.

## Materials and Methods

### Animals and Blood Samples

**Figure 1:**
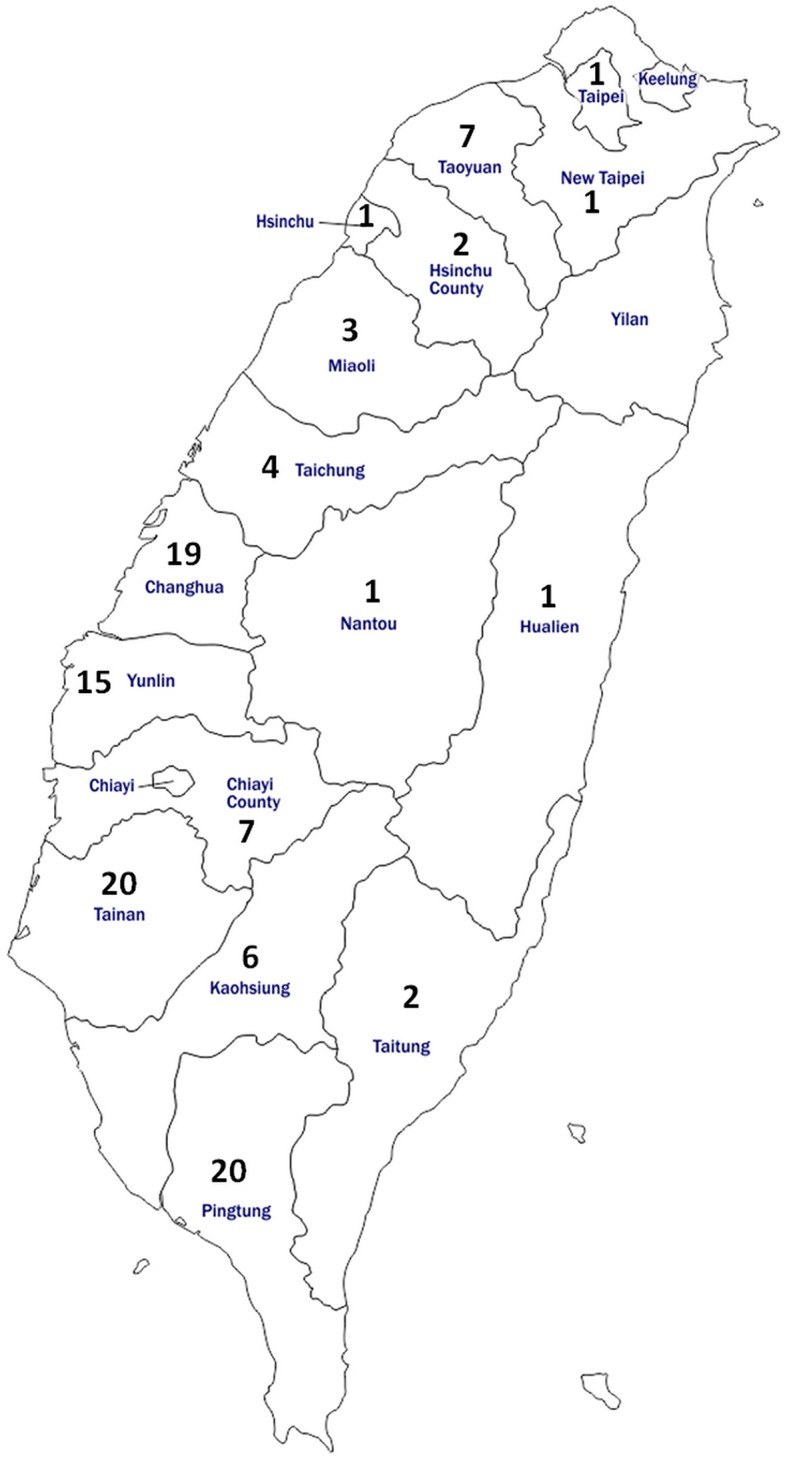
Map of Taiwan showing the location and number of cattle enrolled for anti-BLV antibody detection in the study. In total, 660 bovine serum samples were obtained from 110 herds located in 16 districts of Taiwan.

For the serology test, 660 bovine blood samples were collected from 110 herds that account for 16.7–25.0% of the total herd numbers in each of the 16 cities/counties of Taiwan during the years 2016–2017 (Table 1). Specifically, six healthy cattle without notable abnormality were randomly selected from each of the representative herds. This sampling strategy (six cattle per herd) enabled the detection of at least one BLV-positive animal with 95% confidence at herd-level with an expected seroprevalence of 40%, which was based on the prevalence of Asian countries near Taiwan (17), in the average herd size of 200 cattle. The condition of healthy cows was evaluated on the basis of the general appearance, spirit, appetite, as well as considered the daily milk yield by experienced veterinarians during routine farm visits. The locations and the number of herds analyzed in each district were illustrated in Figure 1. Moreover, detailed information on the sampling for the serology test is provided in Supplementary Table 1.

**Table 1:** The BLV seroprevalence in each city/county.

**Region**	**City/county (districts)**	**Herds examined**	**Positive herds**	**Herd prevalence (%)**	**Animal prevalence (%)**	**Animal prevalence by region (%)**
Northern	Taipei City	1	1	100.0	100.0	91.7
	New Taipei City	1	1	100.0	100.0	
	Taoyuan City	7	7	100.0	95.2	
	Hsinchu City	1	1	100.0	66.7	
	Hsinchu County	2	2	100.0	83.3	
Central	Miaoli County	3	3	100.0	72.2	85.2
	Taichung City	4	4	100.0	91.7	
	Changhua County	19	19	100.0	85.1	
	Nantou County	1	1	100.0	100.0	
Southern	Yunlin County	15	15	100.0	84.4	79.2
	Chiayi County	7	7	100.0	95.2	
	Tainan City	20	20	100.0	81.7	
	Kaohsiung City	6	6	100.0	69.4	
	Pingtung County	20	19	95.0	70.0	
Eastern	Hualien County	1	1	100.0	100.0	72.2
	Taitung County	2	2	100.0	58.3	
	Total	110	109	99.1	81.8	

Moreover, to reveal the correlation between complete blood counts (CBC) and the presence of BLV provirus, another set of 152 blood samples was collected from six herds in five cities (the number of bovine samples of each herd was listed in Table 2). All sampled cows were in healthy conditions under the same criteria as those used for the serologic tests. The blood samples were collected in tubes with anticoagulant, K_2_EDTA, and total DNA was then extracted for PCR amplification.

**Table 2:** The prevalence of BLV proviral DNA in each city/county.

**Region**	**City/county (districts)**	**Herds examined**	**Herd prevalence (%)**	**Animal examined**	**Positive animal**	**Animal prevalence (%)**
Central	Changhua County	2	100.0	48	44	91.7
Southern	Chiayi County	1	100.0	17	17	100.0
	Tainan City	1	100.0	27	23	85.2
	Pingtung County	1	100.0	31	26	83.9
Eastern	Hualien County	1	100.0	29	22	75.9
	Total	6	100	152	132	86.8

The use of animals and experiment protocol were exempt from ethics approval since the samples used in this study were the remaining specimens spared from the Veterinary Medical Teaching Hospital of National Chung Hsing University. Blood samples were collected by experienced large animal veterinarians during routine farm visits for the examination of blood and serum biochemistry, and the use of these specimens for detection of BLV infection was granted by the owners of these private farms.

### Serological Tests by Enzyme-Linked Immunosorbent Assay (ELISA)

BLV gp51 antibody was detected using an IDEXX Leukosis Serum Screening Antibody Test (IDEXX Laboratories, US). Bovine serum samples, both positive and negative controls, as supplied with each kit, were initially diluted 1:20 with the dilution buffer, and the assay was then conducted and analyzed per the manufacturers' instructions. The optical density values of the samples were measured by Sunrise™ (Tecan, Switzerland) at 450 nm.

### Complete Blood Count (CBC)

Analysis of the CBC, including the red blood cell count (RBC), hematocrit (HCT), hemoglobin (HGB), mean cell volume (MCV), mean cell hemoglobin (MCH), mean cell hemoglobin concentration (MCHC), red blood cell distribution width (RDW), white blood cell count (WBC), neutrophil (NEU), lymphocyte (LYM), monocyte (MONO), eosinophil (EOS), basophil (BASO), and platelets (PLT) were conducted using ProCyte Dx™ (IDEXX, USA). The reference intervals of each item used in this study were provided by ProCyte Dx.

### DNA Extraction

Buffy-coat samples were isolated from the anticoagulated blood samples and stored at −20°C for further uses. Genomic DNA was extracted from 5 μL aliquots using the QIAamp DNeasy Blood and Tissue Kit (QIAGEN, Germany) according to the manufacturer's instructions. The DNA was eluted in 200 μL buffer AE, quantified, and stored at −80°C until PCR was performed.

### Detection of BLV Proviral DNA by PCR

BLV partial *env* gene was amplified by nested PCR using two sets of primers, including the outer primer pair (forward primer (BLV-env-1) 5′- TCTGTGCCAAGTCTCCCAGATA−3′ and reverse primer (BLV-env-2) 5′- AACAACAACCTCTGGGAAGGG−3′), resulting in an amplicon 598 bp long, and the inner primer pair (forward primer (BLV-env-3) 5′- CCCACAAGGGCGGCGCCGGTTT−3′ and reverse primer (BLV-env-4) 5′- GCGAGGCCGGGTCCAGAGCTGG−3′), which yielded a fragment 444 bp long (corresponding to 5099-5542 nucleotides of BLV genome of strain K02120). Thermocycler conditions of BLV *env* amplification followed those described in the previous report (13). PCR amplicons were purified using the QIAquick Gel Extraction Kit and then sequenced (Mission Biotec, Taiwan).

### Phylogenetic Analysis of the BLVs

For use in analyzing phylogenetic relationships, in each of the six herds, DNA samples obtained from five cows, which were detected with a higher BLV proviral DNA level, were tested. Thirty DNA amplicons resulted from the first run of PCR were individually isolated and subjected to automated sequencing. In total, 18 distinct sequences of partial *env* gene identified from this study, including the positive control (stains KY419099) and others (accession numbers: MN167071-MN167099), as well as 51 strains representing the 10 genotypes of BLV, were analyzed using MEGA7 software (18), with the neighbor-joining method (19). Detailed information on these viral strains identified from Taiwan and worldwide are summarized in Supplementary Tables 2, 3, respectively.

### Statistical Analysis

The animal- and herd-level seroprevalence were estimated. Kruskal–Wallis tests were used to compare the median animal-level seroprevalence in the districts. A *p*-value of <0.05 indicates a significant difference between the outcome of the independent groups. Simple logistic regression model analysis was used to compare the predictor variable (each item in CBC test) and the outcome variable (results of PCR). The receiver operating characteristic (ROC) curve was used to predict the sensitivity and specificity of each cut-off value, which was used to indicate the presence of BLV proviral DNA. Furthermore, the area under the ROC curve (AUC) was used to compare the combination of sensitivity and specificity among the different categories of the study subjects. All the statistical analyses were carried out in SPSS® statistical version 24 for Mac.

## Results

### Seroprevalence of BLV

Of the 110 herds enrolled in this study, the prevalence was 99.1% (109/110); only one farm located in Pingtung County was detected as BLV negative (Table 1). Moreover, 81.8% (540/660) of cattle were positive among the 660 cattle analyzed. Regionally, positive animals were identified in 91.7% (66/72) of the northern, 85.2% (138/162) of the central, 79.2% (323/408) of the southern, and 72.2% (13/18) of the east coast regions; however, the difference in the BLV seroprevalence among the four regions was not significant (*p* = 0.428).

### Detection of BLV *env* Proviral DNA

Next, 152 plasma samples obtained from apparently healthy cows of six representative herds were examined for the prevalence of BLV proviral DNA by nested PCR. As shown in Supplementary Figure 1, an amplicon with an expected size of 444 bp was amplified from most of the samples, and a positive rate as high as 86.8% (132/152) was observed (Table 2). Of note, 30 out of the 132 BLV-positive samples harbors a higher number of copies of the proviral DNA, which enabled the yield of a 598 bp amplicon in the first run of PCR.

### Phylogenetic Analysis and Sequence Alignment

**Figure 2:**
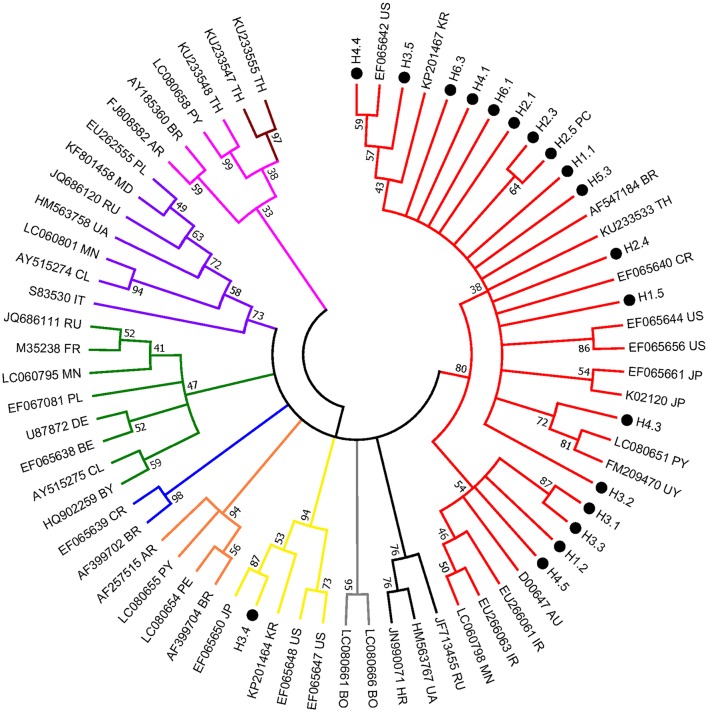
Condensed neighbor-joining phylogenetic tree based on 442 bp of *env* gene sequences of BLV isolates. Taiwanese isolates identified from this study are shown by •, indicating the herd and individual number (e.g., H3.4) (detailed information is included in Supplementary Table 2). The sequences that are 100% identical to the positive control (i.e., H2.5) are not shown. Other representative viral strains indicating the 10 genotypes of BLV (refer to Supplementary Table 3) in the tree are shown by accession number and country of origin. US, United States of America; AR, Argentina; BO, Bolivia; BR, Brazil; CL, Chile; CR, Costa-Rica; PE, Peru; PY, Paraguay; UY, Uruguay; AU, Australia; IR, Iran; JP, Japan; KR, Korea; MN, Mongolia; TH, Thailand; RU, Russia; BE, Belgium; BY, Belarus; DE, Germany; FR, France; HR, Croatia; IT, Italy; MD, Moldova; PL, Poland; UA, Ukraine. Numbers at the branches show bootstrap support (20) (1,000 replicates). Genotypes are indicated by color and labeled at the branches.

The *env* partial sequences of the 30 animals with a much higher level of BLV proviral DNA were identified and then used for molecular analysis; overall, the similarity in the nucleic acid between our local isolates was 100–96.6%. Of note, isolate H3.4 shared the lowest similarity of 96.6% with the sequences of others, and 11 isolates shared identical sequences with the positive control samples (namely, H2.5). The isolates (a total of 19) with distinct sequences were selected for phylogenetic analysis. Consistently, H3.4 is classified into a clade (composed of strains of genotype 3) distant from the other local isolates, which were classified into genotype 1 of BLV) (Figure 2).

**Figure 3:**
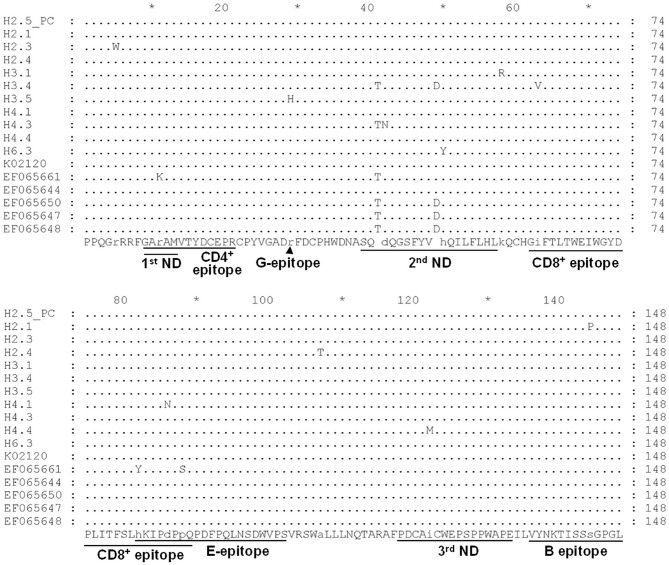
Alignment of deduced partial amino acid sequence of the BLV gp51 protein encoded by the *env* gene from strains in Taiwan. Eleven typical partial gp51 sequences of our local BLV strains, including H2.5 (positive control, PC), H2.1, H2.3, H2.4, H3.1, H3.4, H3.5, H4.1, H4.3, H4.4, and H6.3, were aligned with sequences of reference strains of genotype 1 (K02120, EF065661, and EF065644) and genotype 3 (EF065650, EF065647, and EF065648). The first (1st), second (2nd), and third (3rd) neutralizing domains (ND) and other epitopes were shown at the bottom of the alignment. Dots indicate identical sequences among all the strains. BLV strains were shown by the sample ID.

Noticeably, sequences of H3.4 shares high similarity with those identified from Japan (e.g., 99% with EF065650), Korea (98% with KP201464), and the United States (98% with EF065648). Moreover, alignment of the deduced amino acid sequences demonstrated that the 11 typical local strains share high similarities with those of the corresponding genotypes, although variations sporadically spread out at the middle region of gp51 protein (encoded by env gene) (Figure 3). Previously, several immunogenic regions were identified within this region including three neutralizing domains (ND), a portion of the CD4^+^ T-cell epitope and CD8^+^ T-cell epitope, as well as the viral G, E, and B epitopes (21). Overall, many of the local strains contain single residue substitution at these defined immunogenic regions; for instance, the genotype 3 isolate H3.4 had one amino acid substitution at CD8^+^ epitope, while H3.5 was the only strain with a mutation at G-epitope (Figure 3).

### Correlation of Total WBC Counts and Prevalence of Proviral DNA

**Figure 4:**
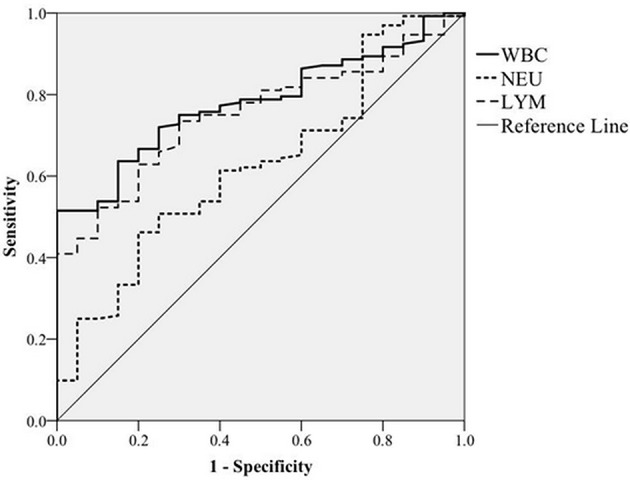
ROC curve analysis. The correlation of white blood cells (WBC), neutrophils (NEU), and lymphocytes (LYM) counts with BLV proviral DNA prevalence detected by PCR was plotted based on receiver operating characteristic curve analysis.

To facilitate control of BLV transmission, an attempt was also made to determine whether total WBC counts could serve as a hematologic marker for monitoring BLV infections. To do so, we analyzed the correlation of total WBC counts with the presence of proviral DNA. In this regard, on the basis of the presence or absence of BLV-proviral DNA, the 152 cattle subjected to proviral DNA detection were divided into two groups. As shown in Table 3, among the parameters of CBC measurement, an increase in WBC, LYM, and NEU are the three predictor variables significantly associated with the presence of BLV-proviral DNA. ROC curve analysis was performed to investigate the correlation between the three parameters and BLV-proviral DNA status. The AUC was similar among the WBC counts (0.78), LYM (0.752), and NEU (0.627) (Figure 4). As the cut-off value was set at 10,215 WBC/μL, the sensitivity and specificity are 72.0 and 75.0%, respectively. Moreover, a value of 12,505 WBC/μL of blood was the best cutoff to differentiate BLV-positive from non-BLV-positive animals, with 51.5% sensitivity and 100% specificity. Furthermore, lymphocyte counts ≥4,855 LYM/μL showed 73.5% sensitivity and 70.0% specificity.

**Table 3:** Correlation among the hematological parameters and the BLV proviral DNA by univariate logistic regression analysis.

**Predictor variable**	**Coefficient**	**S.E.^a^**	***P***	**Exp.^b^ (95% CI^c^)**
RBC	−0.452	0.372	0.224	0.637 (0.307–1.319)
HCT	−0.056	0.060	0.276	0.937 (0.833–1.054)
HGB	−0.339	0.214	0.114	0.713 (0.468–1.085)
MCV	0.012	0.053	0.815	1.012 (0.913–1.122)
MCH	−0.100	0.180	0.578	0.904 (0.635–1.288)
MCHC	−0.386	0.204	0.058	0.680 (0.456–1.013)
RDW	−0.031	0.116	0.791	0.970 (0.773–1.217)
WBC	0.320	0.099	**0.001**	1.378 (1.136–1.671)
NEU	0.355	0.166	**0.033**	1.426 (1.029–1.975)
LYM	0.390	0.138	**0.005**	1.477 (1.126–1.936)
MONO	0.144	0.261	0.582	1.155 (0.692–1.927)
EOS	0.192	0.643	0.765	1.212 (0.343–4.275)
BASO	54.432	36.591	0.137	4.362 × 10^23^ (0–6.108 × 10^54^)
PLT	−0.002	0.002	0.291	0.998 (0.995–1.001)

a *Standard error*.

b *Exponentiation of the coefficient*.

c *95% CI, 95% confidence interval. The bold values indicated the p <0.05*.

## Discussion

In the present study, BLV infection in dairy cows was monitored by the detection of both viral-specific antibody and proviral DNA. This is the first nationwide surveillance of BLV infection in Taiwan since the last study conducted in 1985–1986, and the genotype of BLV was also revealed for the first time.

Apparently, the herd (99.1%) and individual (81.8%) seroprevalences of BLV were remarkably higher than those documented in the previous study, where the values were as low as 8.4 and 5.8%, respectively, in the samples collected in 1985–1986 (16). Despite BLV having been eradicated in some European countries (3), reports from other continents clearly have shown a gradual increase in BLV prevalence over the years, including the United States (22) and Japan (4). Additionally, variations in the BLV prevalence rate were also noticed (23–25). Surprisingly, the BLV infection rate within the herd (81.8%) in Taiwan is only lower than that reported in the United States (83.9%) (22).

The high prevalence indicates BLV is a commonly circulating pathogen and might be spread via multiple routes and by different means. Generally, horizontal transmission is a major means for herds to acquire BLV (26). In Taiwan, the warm and humid subtropical climate could favor the expansion of blood-sucking insect populations (27, 28), the vectors of BLV transmission, which facilitate viral spread. Moreover, our dairy cows are predominantly kept in a loose housing system, and one previous report indicated that this farming system allows frequent contact between animals and possibly leads to an increase in horizontal transmission within a herd (29). Furthermore, although the prevention of iatrogenic transmission has been a common approach for the control of infectious diseases, iatrogenic transmission still accounts for a common means of BLV spread (26). Apparently, an insufficient number of farm animal veterinarians is an issue worldwide (30), including in Taiwan. Based on the document from the Executive Yuan of Taiwan, in 2016, the estimated number of herds was 553, with a national total of 0.1 million dairy cattle. However, the ~30–40 veterinarians who are in farm service cannot meet the demands of current livestock sectors, and farmers often contact veterinarians only when encountering a major problem or for emergencies. Therefore, farmworkers likely manage their flock without consulting veterinarians. As BLV activities have been detected in saliva, milk, and the nasal secretions of cattle (5, 31), in these circumstances, without necessary precautions being taken during cattle handling, processing, and routine husbandry, the iatrogenic transmission would not be avoided. On the other hand, although the possibility of vertical transmission is far less than horizontal, when BLV DNA intermediate as a provirus was integrated into the chromosome of lymphocytes or frozen semen samples (32–35), the offspring might vertically acquire the BLV genome from a parent via *in utero* route or artificial insemination procedure, respectively. Hence, effective BLV surveillance, segregation of BLV-positive cows, and good management practices are essential to minimize BLV transmission.

In the current study, two genotypes of BLV were detected among the 30 samples and only one (sample ID: H3.4) was defined as genotype 3, which indicated genotype 1 is much more predominant than genotype 3. Consistently, it has been shown that genotype 1 is the most prevalent genotype worldwide that has been found in more than 10 countries, including, Korea, Japan, USA, Costa Rica, Argentina, Uruguay, Brazil, Iran, Australia, and Germany (36, 37). While genotype 3 was mainly identified in East Asian countries (Japan and Korea) and North America (USA) (36). Notably, genotype 3 is frequently detected in countries with circulating genotype 1 (36) and that is in concert with our findings.

Despite the detection of proviral DNA, which provides strong evidence of BLV infection, this approach to monitoring is relatively time-consuming and is a more delicate technique than regular hematology analysis. Hence, ideally, total WBC counts could serve as a substitute for DNA detection, especially for on-site diagnosis. Analysis of the CBC indicated an increase in the WBC, NEU, and LYM counts in a large proportion of BLV-positive samples. Of note, in the present study, all samples were collected from cows without clinical signs of illness, indicating that BLV infection can affect the bovine immune system without the animal showing clinical symptoms. Moreover, as indicated in Table 3, elevated WBC, LYM, and NEU counts in dairy cattle were significantly correlated with the presence of BLV proviral DNA. Nevertheless, based on the ROC analysis (Figure 4), the AUC of the WBC and LYM counts were ~0.7–0.8, indicting acceptable accuracy for the diagnosis of BLV infection. In a similar study, Alvarez and colleagues quantified proviral DNA in blood samples and divided the subjects into two categories based on the BLV DNA levels: i.e., undetectable to low (aleukemic stage) and high (lymphocytic stage) (38). The results indicate that a cutoff value of 13,400 WBC/μL of blood could differentiate aleukemic from leukemic cattle, with 86.6% sensitivity and 80.35% specificity (38); the AUC of 0.911 indicates good discrimination by this method. However, despite the accumulated evidence that supports the infection level of BLV being reflected in the WBC counts, a survey with a large sample size is necessary to definitively establish WBC counts as an independent predictor of BLV infection. Or in addition to hematologic evidence, other clinical indexes, in particular, fever, swollen lymph nodes, poor appetite, and milk quality, could also be considered as markers for the potential of BLV transmission among cattle.

In conclusion, apparently, BLV infection is highly endemic in Taiwan, where a sustainable strategy to manage BLV infection in cattle herds is still lacking. Hence, the major risk factors involved in BLV transmission first should be identified, and on the basis of those results, a comprehensive program to prevent BLV transmission could be implemented to control new infections.

## Data Availability Statement

The data in this study has been deposited to the GenBank database (https://www.ncbi.nlm.nih.gov/genbank/) using the accession numbers: MN167071, MN167072, MN167073, MN167074, MN167075, MN167076, MN167077, MN167078, MN167079, MN167080, MN167081, MN167082, MN167083, MN167084, MN167085, MN167086, MN167087, MN167088, MN167089, MN167090, MN167091, MN167092, MN167093, MN167094, MN167095, MN167096, MN167097, MN167098, MN167099.

## Ethics Statement

Ethical review and approval was not required for the animal study because the samples used in this study were remaining specimens spared from Veterinary Medical Teaching Hospital of National Chung Hsing University. The use of these specimens for detection of BLV infection was granted by the owners of the animals.

## Author Contributions

S-TC designed the experiment and analyzed data. J-CH and C-YL conducted the experiments and data analysis. W-LH analyzed data and wrote the manuscript.

### Conflict of Interest

The authors declare that the research was conducted in the absence of any commercial or financial relationships that could be construed as a potential conflict of interest.

## Supplementary Material

Supplementary Figure 1 Detection of BLV *env* gene by nested-PCR. Total DNA served as the template for the amplification of the BLV *env* gene. After the second run of PCR, the amplicon with an expected size of 444 bp was yielded from some of the samples. L, 100 bp DNA ladder. N, negative control. P, positive control. 1–9, samples number 1–9.

Supplementary Table 1 Information of the samples in the investigation of seroprevalence of BLV infection in Taiwan.

Supplementary Table 2 Information of the representative sequences identified in Taiwan that were used for phylogenetic analysis.

Supplementary Table 3 The selected strains/isolates representing the 10 genotypes of BLV used for phylogenetic analysis.
