# Supplementary figures and images for: Molecular Epidemiological and Serological Studies of Bovine Leukemia Virus in Taiwan Dairy Cattle
Source: Front Vet Sci. 2019 Dec 6;6:427. doi: 10.3389/fvets.2019.00427 (PMC6908947; doi:10.3389/fvets.2019.00427)

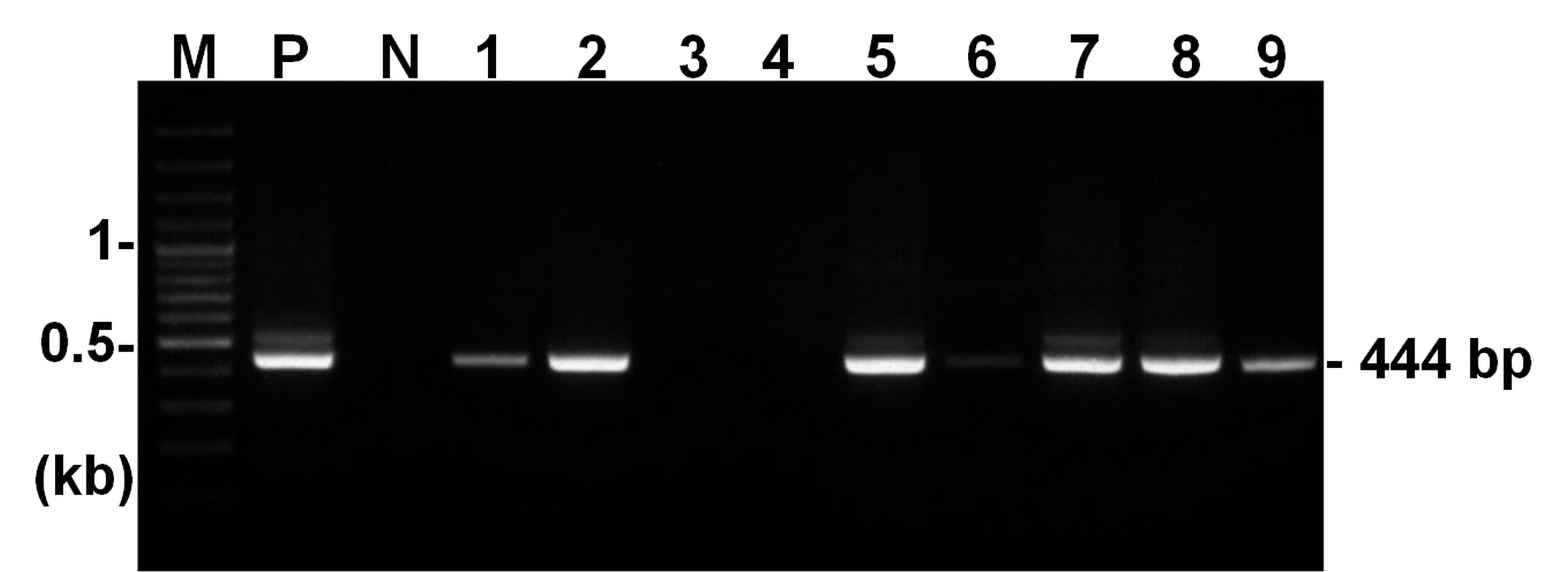

Supplement: Supplementary Figure 1 — Detection of BLV env gene by nested-PCR. Total DNA served as the template for the amplification of the BLV env gene. After the second run of PCR, the amplicon with an expected size of 444 bp was yielded from some of the samples. L, 100 bp DNA ladder. N, negative control. P, positive control. 1–9, samples number 1–9. [file Image_1.JPEG]
