# Supplementary material for: Molecular Epidemiological and Serological Studies of Bovine Leukemia Virus in Taiwan Dairy Cattle
Source: Front Vet Sci. 2019 Dec 6;6:427. doi: 10.3389/fvets.2019.00427 (PMC6908947; doi:10.3389/fvets.2019.00427)
Supplement: Supplementary Table 1 — Information of the samples in the investigation of seroprevalence of BLV infection in Taiwan. [file Data_Sheet_1.PDF]

Supplementary Table S1. Information of the samples in the investigation of seroprevalence of BLV infection in Taiwan.

**Region**	**District** (city/county)	Herds in each district	Herds analyzed	Percentage of herds analyzed in each city/county
Northern	Taipei City	1	1	100.0%
	New Taipei City	6	1	16.7%
	Taoyuan City	33	7	21.2%
	Hsinchu City	4	1	25.0%
	Hsinchu County	8	2	25.0%
Central	Miaoli County	16	3	18.8%
	Taichung City	20	4	20.0%
	Changhua County	97	19	19.6%
	Nantou County	5	1	20.0%
Southern	Yunlin County	73	15	20.5%
	Chiayi County	35	7	20.0%
	Tainan City	104	20	19.2%
	Kaohsiung City	32	6	18.8%
	Pingtung County	99	20	20.2%
Eastern	Hualien County	5	1	20.0%
	Taitung County	8	2	25.0%

Supplementary Table S2. Information of the representative sequences identified in Taiwan that were used for phylogenetic analysis.

Isolates in Taiwan	Accession number	Abbreviation
2016NCHU001	MN167071	H1.1
2016NCHU002	MN167072	H1.2
2016NCHU003	MN167073	H1.3
2016NCHU004	MN167074	H1.4
2016NCHU005	MN167075	H1.5
2016NCHU006	MN167076	H2.1
2016NCHU007	MN167077	H2.2
2016NCHU008	MN167078	H2.3
2016NCHU009	MN167079	H2.4
2016NCHU	KY419099	H2.5*
2016NCHU010	MN167080	H3.1
2016NCHU011	MN167081	H3.2
2016NCHU012	MN167082	H3.3
2016NCHU013	MN167083	H3.4
2016NCHU014	MN167084	H3.5
2016NCHU015	MN167085	H4.1
2016NCHU016	MN167086	H4.2
2016NCHU017	MN167087	H4.3
2016NCHU018	MN167088	H4.4
2016NCHU019	MN167089	H4.5
2016NCHU020	MN167090	H5.1
2016NCHU021	MN167091	H5.2
2016NCHU022	MN167092	H5.3
2016NCHU023	MN167093	H5.4
2016NCHU024	MN167094	H5.5
2016NCHU025	MN167095	H6.1
2016NCHU026	MN167096	H6.2
2016NCHU027	MN167097	H6.3
2016NCHU028	MN167098	H6.4
2016NCHU029	MN167099	H6.5

* the field sample served as positive control for PCR reaction.

Supplementary Table S3. The selected strains/isolates representing the 10 genotypes of BLV used for phylogenetic analysis.

Genotype	Strain/Isolate	Year	Country of origin	Accession number
1	-	1990[a]	Australia	D00647
	141	2004[b]	Brazil	AF547184
	CRAG-2	2016[b]	Costa-Rica	EF065640
	Isfuhan	2007[b]	Iran	EU266061
	Thehran	2007[b]	Iran	EU266063
	JPMI-2	2004[a]	Japan	EF065661
	λ-BLV	1985[a]	Japan	K02120
	BLV_Mongolia-7	2014[a]	Mongolia	LC060798
	asun1	2007[a]	Paraguay	LC080651
	GBGS-10	2014[a]	South Korea	KP201467
	Lb11-B5	2013[a]	Thailand	KU233533
	Uru33	2008[b]	Uruguay	FM209470
	USIA	1975[a]	USA	EF065644
	USPA	1976[a]	USA	EF065656
	USWI	1991[a]	USA	EF065642
2	B19	2000[b]	Argentina	AF257515
	384	2004[b]	Brazil	AF399704
	par17	2007[a]	Paraguay	LC080655
	lima40	2008[a]	Peru	LC080654
3	JFPU	2004[a]	Japan	EF065650
	GBGS-11	2014[a]	South Korea	KP201464
	USCA-1	1997[a]	USA	EF065647
	USCA-2	1997[a]	USA	EF065648
4	2_BY	2013[b]	Belarus	HQ902259

	BG	2006[b]	Belgium	EF065638
	145	2005[b]	Chile	AY515275
	LB59	1990[a]	France	M35238
	3	1997[b]	Germany	U87872
	BLV_Mongolia-4	2014[a]	Mongolia	LC060795
	PL1	2016[b]	Poland	AF067081
	NV13	2010[a]	Russia	JQ686111
5	8513	2004[b]	Brazil	AF399702
	CRGC	2006[b]	Costa-Rica	EF065639
6	PL-1238	2009[b]	Argentina	FJ808582
	151	2004[b]	Brazil	AY185360
	par91	2007[a]	Paraguay	LC080658
	Pa53-A4	2013[a]	Thailand	KU233548
7	14	2005[b]	Chile	AY515274
	I2	1996[b]	Italy	S83530
	16MD	2012[a]	Moldova	KF801458
	BLV_Mongolia-10	2014[a]	Mongolia	LC060801
	Australian type 151	2016[b]	Poland	EU262555
	NK17	2010[a]	Russia	JQ686120
	3-15	2016[b]	Ukraine	HM563758
8	ELG_Cro/ORA/09	2009[a]	Croatia	JN990071
	N174	2009[a]	Russia	JF713455
	3-43	2013[b]	Ukraine	HM563767
9	mon17	2008[a]	Bolivia	LC080660
	mon22	2008[a]	Bolivia	LC080661
10	Pa51-A3	2013[a]	Thailand	KU233547

	Pr67-C5	2013[a]	Thailand	KU233555

[a] Year of collection. [b] Year of paper published.
